# The potential yield of Tai Chi in cancer survivorship

**DOI:** 10.4155/fsoa-2016-0049

**Published:** 2016-10-20

**Authors:** Lee Smith, Dan Gordon, Adrian Scruton, Lin Yang

**Affiliations:** 1 Department of Life Science, The Cambridge Centre for Sport & Exercise Sciences, Anglia Ruskin University, Cambridge, UK; 2 Department of Epidemiology, Center for Public Health, Medical University of Vienna, Austria

**Keywords:** cancer survivorship, cognitive function, light intensity physical activity, physical fitness, psychological health, Tai Chi

The purpose of the current paper is to encourage research into all areas of Tai Chi and cancer survivorship. Tai Chi is defined here as a combination of Chinese philosophy, martial and healing arts. Tai Chi is a form of physical activity that is carried out at either a light or moderate intensity. The practice of Tai Chi integrates mental concentration and breathing control [1,2]. We first discuss the role of light physical activity in cancer survivorship and then narrow our focus to Tai Chi *per se*.

## Cancer survivorship

From the point of clinical cancer diagnosis one may be categorized as a cancer survivor. There are approximately 14.1 million cancer survivors, across all cancer types, worldwide and this figure is expected to rise to 24 million by 2035 [3]. This gradual rise in the prevalence of cancer survivors is likely owing to the aging population and advancements in cancer treatment. The first and fourth most common cancers in the UK are breast and colorectal, respectively. Prostate cancer is the most common cancer in UK men. Approximately 80% of patients survive breast cancer for at least 10 years. Some 59 and 85% of colorectal and prostate cancer patients, respectively, survive 5 years or more. However, approximately 20, 20–30 and 40–60% of breast, prostate and colorectal cancer survivors, respectively, will experience cancer recurrence [4].

## Cancer survivorship & physical activity

Regular physical activity (defined as any bodily movement produced by skeletal muscle that requires energy expenditure) [5] is associated with a reduced risk of developing cancer and of recurrence of breast, prostate and colorectal cancer, and with improved survival. Such benefits are putatively mediated by reductions in adiposity, with resulting changes to circulating adipokines and cytokine, insulin resistance and blood insulin levels, and sex hormone production; increased insulin sensitivity in skeletal muscle (and thus reduced hyperinsulinemia); increased colonic motility, leading to increased transit time (and thus reduced colonic carcinogen exposure); reduced DNA oxidative damage and increased repair [6–9]. In 2010, the American College of Sports Medicine convened a round table (which included cancer survivors) to review the safety and efficacy of exercise training during and after cancer treatment, and to provide guidelines. It concluded that cancer survivors should follow the physical activity guidelines for Americans with specific exercise programming adaptations based on disease- and treatment-related adverse effects. Similar guidelines have been produced by The British Association of Sport and Exercise Sciences [10]. However, despite such benefits and the availability of guidelines, physical activity levels in these populations are low during and after treatment [11]. Interventions to promote physical activity in these groups are urgently needed and more research is required to inform such interventions.

A recent systematic review of controlled trials of physical activity in cancer survivors identified 16 interventions to increase physical activity levels post treatment, of which only six demonstrated statistically significant impacts [12]. Likewise, of 14 trials of such interventions subjected to a Cochrane review, none of the included trials reported that 75% or greater adherence of the intervention group met current physical activity guidelines at follow-up [13]. However, those interventions to promote exercise in cancer survivors, which were associated with greatest adherence, shared some common features: the setting of program goals, promoting practice and self-monitoring, and encouraging participants to attempt to generalize behaviors learned in supervised exercise environments to other, nonsupervised contexts. One such means by which to apply such learning is through group exercise. Little research exists on group exercise in cancer survivors and this is an area that needs further exploration.

A key barrier to physical activity participation in cancer survivors is the sensation of debilitating fatigue. However, meta-analytic data from randomized controlled trials of physical activity in cancer survivors of mixed diagnoses (but mostly breast cancer) show improvements in many outcomes that are of particular importance to patients, including reductions in cancer-related fatigue [12]. This presents physical activity practitioners with a conundrum, in that fatigue is a barrier to physical activity participation but indeed physical activity participation reduces fatigue. Novel approaches to overcome fatigue through physical activity participation in cancer survivors are needed. Physical activity energy expenditure (PAEE) may be defined as energy expended above that of the resting level. Previous work has shown prospective associations between PAEE and various health outcomes, including metabolic risk. Smith *et al*. [14] have proposed that increasing levels of PAEE through standing or light activities (e.g., incidental movement) have desirable health effects equivalent to those of a moderate-to-vigorous intensity. Indeed sedentary behavior has been associated with an increased risk of developing primary cancer, independent of physical activity levels; interventions that promote levels of PAEE through light activity may be more successful than those that target only moderate-to-vigorous physical activity. Cancer survivors may be more willing to replace sedentary activities with standing or light activities, rather than activities of moderate intensity, as there are fewer potential barriers (e.g., motivation to undertake light activity typically requires lower cognitive effort and elicits less palpable physiological responses). One such means to increase physical activity energy expenditure through light group-based exercise is Tai Chi.

## Cancer survivorship & Tai Chi

Tai Chi (also spelled as ‘Tai Chi Chuan’ or ‘Taiji’), a form of Qigong, is an exercise that originated in ancient China and is a combination of Chinese philosophy, martial and healing arts [1]. The Compendium of Physical Activities assigned a Metabolic Equivalent of task score of 4.0 to Tai Chi, which is classified as moderate intensity activity [15]. Yet in older adults and adults with health conditions, Tai Chi is frequently carried out in a gentle and nonstrenuous form (light-intensity physical activity) making it suitable for all ages, fitness levels and potentially cancer survivors. Moreover, compared with other forms of Qigong, Tai Chi is better suited for cancer survivors, owing to the simplicity of the movements involved with the exercise. Such simplicity allows those previously unfamiliar with Tai Chi to carry out the activity with ease and confidence.

## Tai Chi, physical & psychological health

To date, Tai Chi has shown feasibility and improved physical and psychological outcomes among older adults, patients with chronic conditions and breast cancer survivors. A recent meta-analysis synthesized randomized controlled trial (RCT) on the effect of Tai Chi on treatment-related outcomes in breast cancer survivors [16]. A total of nine RCTs carried out in the USA were included. The meta-analysis reported significant improvements in handgrip dynamometer strength (p = 0.001) and physical fitness (p ≤ 0.001) in three studies, and improved physical well-being (p = 0.07) in five studies. Two RCTs (n = 19 and 21, respectively) in breast cancer survivors measured disease-specific biomarkers and reported marginally significant improvements in circulating IL-6 and IGF-1 (p = 0.05 and 0.06, respectively) [16]. The meta-analysis showed that RCTs targeting Tai Chi in breast cancer survivors have been limited by small samples (range n = 21–55, with one trial n = 90), short follow-ups (only 1/6 collected data beyond 12 weeks) and inconsistent biomarkers [16]. Future RCTs in breast cancer survivors investigating the health benefits of Tai Chi should address such limitations. One trial carried out in the USA randomly assigned breast cancer survivors (n = 21) to one of two groups receive Tai Chi; or receive psychosocial support; groups received three sessions a week for 12 weeks. Self-reported psychological outcomes were recorded at baseline, 6 and 12 weeks. Significant improvements were found in self-esteem (p = 0.01) in the Tai Chi group compared with the psychosocial support group [17]. Studies in other cancer sites are limited and more research in this area is warranted. A recent review on complementary and alternative medicine in cancer survivors of mixed diagnosis identified that participation in Tai Chi had a positive influence on quality of life and psychological health [18]. Importantly, Tai Chi trials in breast cancer survivors have reported high retention rates (82–91%) compared with retention rates below 85% in other exercise promotion trials. Therefore, Tai Chi may be a sustainable form of physical activity for this population. We encourage longitudinal trials with at least 6 months follow-up to test this hypothesis.

## Tai Chi & cognitive function

In addition to the potential benefits on physical fitness and psychological health, Tai Chi may negate the deleterious cognitive side effects of cancer and its treatment. Tai Chi incorporates the practice of meditation [1,2]. This meditative element of Tai Chi might extend its benefits beyond other forms of physical activity. An RCT carried out in the USA in healthy older adults compared changes in cognitive function over a 12 month period. Participants were assigned to one of three conditions: participation in Tai Chi (n = 37); participation in standard exercise (endurance, resistance/strength and flexibility exercise at YMCA, n = 37); and participation in ‘healthy aging’ classes (n = 56). Cognitive function was measured using semantic fluency, and forward and backward digit-span tests at baseline, 6 and 12 months. A significant improvement in backward digit-span in the Tai Chi group compared with other conditions was found at 6 months (p ≤ 0.001), and maintained at 12 months (p = 0.01) [2]. In a study of 23 US women with mild to moderate cognitive impairment a year or more after chemotherapy, taking a 60-min Tai Chi class twice a week for 10 weeks resulted in improved immediate memory, delayed memory, verbal fluency, attention and executive function (all p < 0.05). Improved self-reported cognitive function in verbal (p = 0.01) and visual memory (p < 0.05) measured by the Multiple Abilities Self-Reported Questionnaire, were also reported [19]. This feature of Tai Chi may be beneficial for cancer survivors who are often facing impaired cognitive function from cancer treatment. After chemotherapy treatment for cancer, about a third of cancer survivors will experience ‘chemo brain’. ‘Chemo brain’ describes chemotherapy-related cognitive impairment that results in neuropsychological difficulties following cancer treatment, such as lack of concentration and short-term memory loss [19,20]. This phenomenon is critical, particularly among young adult cancer survivors due to its negative impact on workability, work performance, associated workforce loss and economic burden. Research has identified that impaired cognitive function [20], change in brain metabolism [21] and change in brain structure [22] are associated with cancer treatment; however, the cause of such ‘chemo brain’ phenomenon is unclear. Treatment and therapies for ‘chemo brain’ is thus limited. Further research using experimental design and sophisticated brain imaging technologies (e.g., brain PET, functional MRI) to detect the brain structural and functional outcomes associated with Tai Chi is warranted.

## Summary & recommendations

Tai Chi is accessible to most people and does not require special facilities or expensive equipment. Tai Chi is an understudied but promising tool to increase light physical activity levels with additive meditative benefits in cancer survivors and thus improving survival outcomes (e.g., reduction in cancer recurrence, improved psychosocial health and cognitive function). The mechanisms of such impact and the potential of scaled-up Tai Chi implementation in cancer survivors are largely unknown. Given the likelihood for high acceptability of Tai Chi among this particularly vulnerable population, with their distinctive challenges and the potential positive impact on survival, research is urgently needed to uncover and understand mechanistic pathways for Tai Chi to improve cancer survival and to ultimately become part of routine care. Research is also required on the development of robust Tai Chi interventions for this population (recruitment pathways, length and frequency of Tai Chi classes, enjoyment, among others). We encourage research into all areas of cancer survivorship and Tai Chi.
